# Treatment of Advanced Hepatocellular Carcinoma with Somatostatin Analogues: A Review of the Literature

**DOI:** 10.3390/ijms20194811

**Published:** 2019-09-27

**Authors:** Hendrik Reynaert, Isabelle Colle

**Affiliations:** 1 Department of Gastroenterology-hepatology UZBrussel, Laarbeeklaan 101, 1090 Brussels, Belgium; 2 Liver Cell Biology Lab, Vrije Universiteit Brussel (VUB), Laarbeeklaan 103, 1090 Brussels, Belgium; 3 Department of Gastroenterology-hepatology, ASZ Aalst, Merestraat 80, 9300 Aalst, Belgium; Isabelle.Colle@UGent.be; 4 Department of Gastroenterology-hepatology, Ghent University, De Pintelaan, 9000 Ghent, Belgium

**Keywords:** hepatocellular carcinoma, octreotide, somatostatin, somatostatin analogues, somatostatin receptor

## Abstract

Hepatocellular carcinoma, one of the most dreaded complications of cirrhosis, is a frequent cancer with high mortality. Early primary liver cancer can be treated by surgery or ablation techniques, but advanced hepatocellular carcinoma remains a challenge for clinicians. Most of these patients have underlying cirrhosis, which complicates or even precludes treatment. Therefore, efficacious treatments without major side effects are welcomed. Initial results of treatment of advanced hepatocellular carcinoma with somatostatin analogues were promising, but subsequent trials have resulted in conflicting outcomes. This might be explained by different patient populations, differences in dosage and type of treatment and differences in somatostatin receptor expression in the tumor or surrounding tissue. It has been shown that the expression of somatostatin receptors in the tumor might be of importance to select patients who could benefit from treatment with somatostatin analogues. Moreover, somatostatin receptor expression in hepatocellular carcinoma has been shown to correlate with recurrence, prognosis, and survival. In this review, we will summarize the available data on treatment of primary liver cancer with somatostatin analogues and analyze the current knowledge of somatostatin receptor expression in hepatocellular carcinoma and its possible clinical impact.

## 1. Introduction

According to 2018 statistics of the World Health Organization, liver cancer is the sixth most frequent cancer and the fourth most frequent cause of cancer related death worldwide. The incidence is increasing over the last decades, and the estimated number of deaths due to liver cancer in 2018 was approximately 781,000 (http://gco.iarc.fr/today/home, accessed on April 10, 2019).

Most often, hepatocellular carcinoma (HCC) develops in a cirrhotic liver, in fact 3–8% of cirrhotic patients will develop HCC during their life [1]. The diagnosis of HCC can usually be made by contrast-enhanced computed tomography (CT) or magnetic resonance imaging (MRI), which will also allow to determine the diameter and the number of hepatic lesions, and the presence of portal or hepatic vein thrombosis or invasion. Liver biopsy is rarely needed to confirm the diagnosis if the tumor is > 2 cm. Skeletal and lung metastasis should be excluded by bone scan and thoracic CT scan. Finally, it is important to determine liver function, the presence of portal hypertension and the tumor induced performance status of the patient. Treatment will be selected according to the Modified Barcelona Cancer Liver Clinic (BCLC) staging system, which takes into account all of the above variables. Patients with very early stage BCLC 0 (single tumor < 2 cm, no vascular invasion, good health status Eastern Cooperative Oncology Group (ECOG) 0 and well-preserved liver function) have a good prognosis and can be treated by surgery or ablation techniques. Patients with early stage tumor BCLC A (Single or 2–3 nodules < 3 cm, preserved liver function and ECOG 0) also have a fairly good prognosis when treated by surgery, ablation or transplantation. Patients with BCLC 0 and A are candidates for curative treatments, but unfortunately, only 20% of patients with HCC qualify for these types of treatment. When the stage is more advanced, curative treatments are impossible and survival gradually decreases. Intermediate BCLC stage B (Multinodular tumor that is unresectable, preserved liver function, but still good health status ECOG 0) can be treated by chemoembolization, which augments median survival from 16 to approximately 40 months. Patients with advanced HCC, BCLC stage C, (symptomatic tumors, ECOG 1-2, macrovascular invasion and/or extrahepatic spread) have a poor prognosis, with expected median survival time of 6 to 8 months. With the introduction of effective systemic treatment (Sorafenib), median survival is now around 10 months. Several new effective drugs have been approved recently or will be approved in due and increasing survival in this group of patients can be expected. Sorafenib and Lenvatinib are considered first line therapies, whereas Regorafenib, Cabozantinib and Ramucirumab are second line agents [1,2]. Sorafenib, Lenvatinib and Cabozantinib are oral multi-kinase inhibitors that inhibit the activity of various kinases. Ramucirumab is a recombinant monoclonal human immunoglobulin IgG1 antibody specific for VEGFR-2. Overall, survival of patients with advanced HCC who are treated with these agents is between 10 and 13.6 months [3]. Furthermore, studies with immunotherapy for HCC are promising [3]. Nivolumab and pembrolizumab are recombinant monoclonal human immunoglobulin IgG4 antibodies specific for human programmed cell death protein-1. These immune checkpoint inhibitors block a signal that prevents activated T cells from attacking the cancer, thus allowing the immune system to attack the cancer [4]. Trials studying combination therapies with several immune checkpoint inhibitors and kinase inhibitors are currently being conducted. Preliminary results are promising, but the final results are eagerly awaited. Even if much progress has been made in the last few years, these new treatments are not free from side effects, especially in patients who are in poor general condition, including the patients with cirrhosis and advanced HCC.

Patients with end-stage disease are characterized by very poor performance status (ECOG 3-4) and/or end stage liver function. At this moment, best supportive care is the only option for these patients who have a median survival of 3 to 4 months [1,5]. Therefore, efficient treatments without major side effects are needed for this specific population. More than 20 years ago, initial results of somatostatin (SST) analogue treatment for inoperable HCC were promising, but subsequent trials have resulted in conflicting outcomes. The reason why results were inconsistent is not completely clear, but in all probability treatment as well as patient populations and tumor characteristics were very diverse in the different trials, which can explain inconsistent outcome.

## 2. Molecular basis of Treating HCC with Somatostatin Analogues

Somatostatin is a 14-amino acid polypeptide produced and released by neuroendocrine, inflammatory and immune cells. Somatostatin releasing cells were first described in the hypothalamus, but have been shown to occur in many other organs including the central and peripheral nervous system, pancreas, gut, thyroid, adrenals, spleen, liver, kidneys and prostate [6]. The effects of SST are mediated via somatostatin receptors (SSTRs) of which 5 subtypes, termed SSTR1-5, have been described. The natural SST-14 and SST-28, have nanomolar affinity for all 5 receptors. Octreotide and lanreotide are synthetic SST analogues that are commercially available and approved for human use. They have high affinity for SSTRs 2 and 5, some affinity for SSTR3, but none for SSTR1 and 4 [7]. Pasireotide is a newer synthetic multiligand SST analogue which has very high functional activity on SSTR1, 3 and 5, but low functional activity on SSTR2 [8].

Somatostatin are receptors that are expressed in various normal and neoplastic tissues, of which expression in neuroendocrine tumors is best characterized. Somatostatin receptors are also expressed to varying degrees in solid organ tumors including melanoma and prostate, breast, ovary, thyroid, and gastrointestinal cancers. Reubi et al. were the first authors to demonstrate the presence of SSTRs in HCC [9]. We and others have confirmed several years ago that different subtypes of SSTRs are expressed in human cirrhosis and HCC [10,11,12,13,14,15]. Even if most SSTR subtypes have been shown to be present in HCC, there is high variability in expression in the different studies. Even if most SSTR subtypes have been shown to be present in HCC, there is high variability in the expression in the different studies. The expression of SSTR subtypes 1, 2, 3 and 5 in HCC cell lines and tumor tissue ranged from 46–76%, 30%–67%, 0–64%, and 0–76% respectively [9,10,11,12,13,14,15]. The reason for variability might be differences in tumor biology, but also different techniques to demonstrate SSTR expression. Moreover, SSTR expression was correlated with markers of poor prognosis [11]. Indeed, SSTR2 membrane staining was associated with high AFP levels, poor differentiation, and CK19 expression, all of which are predictors of poor survival.

Somatostatin and its analogues bind to the SSTRs, which are inhibitory G protein-coupled receptors expressed on the membrane of the cell. Activation of the receptors results in activation of ion channels and intracellular enzymes, which activate or inhibit intracellular pathways, which in turn influence tumor cells [16]. One can distinguish direct and indirect actions on tumor cells. Direct effects comprise induction of apoptosis, inhibition of cell cycle, inhibition of migration, and inhibition of growth factor release by tumor cells. Indirect effects include: inhibition of angiogenesis, modulation of immune system, and inhibition of secretion of growth hormones and growth factors [16,17]. The different SSTRs are coupled with different inhibitory G-proteins and selective activation of SSTR subtypes will result in distinct effects. For example, SSTR subtypes 1, 2, and 3 transduce their antiproliferative action by stimulating protein tyrosine phosphatases, which in turn affect the mitogenic mitogen-activated protein kinase; stimulation of SSTR3 induces apoptosis and inhibition of angiogenesis [16]. Some neuroendocrine tumors become resistant to treatment with SST analogues. Several mechanisms have been proposed to explain the decreased or absent response to SST analogues: SSTR downregulation after continued exposure to the agonist; internalization of the SSTR; desensitization, which is decreased response due to receptor uncoupling from second messenger activation; different expression of SSTR in tumors including absence of some SSTRs and/or upregulation of SSTRs that do not recognize the SST analogue [18]. In some instances, resistance occurs even in the presence of the receptor. Indeed, in some neuroendocrine and pituitary tumors, a truncated or spliced SSTR5 variant blocks SSTR2 signaling, which explains the lack of effect of SST analogues in these tumors, even in the presence of normal SSTR2 expression [17].

Currently, a clinically relevant anti-tumor effect of SST analogues has been clearly demonstrated in advanced neuroendocrine tumors and SST analogues are generally accepted as therapy for these tumors [19]. Several anti-tumor effects of SST and SST analogues have been described in HCC. The above reviewed anti-neoplastic effects of SST analogues have also been demonstrated in HCC cell lines, including effects on proliferation, apoptosis, invasion, angiogenesis, and inhibition of secretion of growth hormones and growth factors such as insulin and insulin-like growth factor-1 [10,20,21,22]. From these pre-clinical data and the beneficial effect of SST analogues in the treatment of neuroendocrine tumors, it was proposed that SST analogues could have a role in the management of inoperable HCC.

## 3. Clinical Trials of Treatment of HCC with SST Analogues

In the past 20 years, several clinical trials assessing the effect of SST analogues on tumor progression, survival and quality of life in patients with advanced HCC have been published. It is difficult to compare studies and to draw general conclusions because the set-up of different trials was diverse; some trials were open and not controlled, some were randomized controlled, some compared octreotide to placebo, some added octreotide to another treatment. Moreover, different SST analogues, different formulations (short acting and long acting), and different treatment schedules were used.

The first study, a randomized controlled trial (RCT) performed in 1998, compared subcutaneously (SC) octreotide 250 μg bis in die (BID) versus no treatment in 58 patients (28 in the treatment group, 30 in the control arm) [23]. All patients had biopsy proven HCC; most patients had advanced disease with large and/or multiple tumors. Seventy-seven% had cirrhosis of which 38% had Child-Pugh class B and 56% class C. The results were exciting: Treated patients had a statistically significant increased median survival of 13 months versus 4 months for patients who were not treated. Cumulative survival rate at 6 and 12 months was 75% versus 37%, and 56% versus 13% respectively. In some treated patients, small tumors disappeared or remained unchanged, whereas, the tumor size increased in all non-treated patients. Moreover, more than half of the treated patients reported improved quality of life. A second large RCT that demonstrated survival benefit from octreotide treatment was published in 2007 [24]. The design of this study was interesting. A total of 127 patients, with advanced HCC and cirrhosis Child-Pugh stage A or B, were enrolled in the study. Octreoscan was performed in all patients who were randomized according to the scintigraphy result. Sixty-six patients with a negative Octreoscan received no treatment; of the 61 patients with a positive scintigraphy, 31 were allocated to receive octreotide, 30 to placebo. Treatment schedule was as follows: octreotide 500 μg SC ter in die [25] for 6 weeks; octreotide long-acting release (LAR) 20 mg intramuscularly (IM) at week 4 and 8, and thereafter octreotide LAR 30 mg IM every 4 weeks. Patients in the octreotide group had statistically better median overall survival than patients in the placebo group: 49 weeks versus 28 weeks. Interestingly, patients with SSTR negative scintigraphy had an overall survival of 28 weeks, exactly the same as patients with a positive scintigraphy treated with placebo, suggesting that patients with a SSTR negative HCC do not benefit from treatment with octreotide. Several other, unblinded RCT trials, mainly performed in Asia, showed also some overall survival benefit [26,27,28,29,30]. In all these studies short acting octreotide was given in a dosage of 100–200 μg 2 to 3 times per day; octreotide was compared to no treatment (Table 1). The first trial assessing the value of long acting SST analogues in patients with advanced HCC was published in 2002 [25]. Thirty-two patients with inoperable HCC treated with long acting SST analogues (octreotide LAR 20 mg IM every 4 weeks or lanreotide 30 mg IM every 2 weeks) were compared to 27 untreated historical controls. Quality of life was better in the treated group, tumor was stable and even regressed in 40% of treated patients and overall survival significantly increased from 8 to 15 months. In another non-randomized trial, 63 patients with unresectable HCC were treated with octreotide LAR 20 mg IM every 28 days [31]. All patients underwent octreotide scintigraphy. There was a trend of better median overall survival of patients who had a positive octreotide scintigraphy versus those patients with a negative scan (9.7 versus 6.8 months), but this difference was not statistically significant. In a non-randomized German study, 41 patients were treated with octreotide [32]. The treatment schedule was as follows: octreotide 50 μg SC TID day in the first week, after which the dose was increased each week until a final dose of 250 μg TID, followed by 30 mg octreotide LAR IM every 4 weeks. Most patients had cirrhosis, including 20% Child C, and all patients had inoperable advanced HCC. Median overall survival was 571 days (19 months), which was comparable to the survival of a group of patients treated with transarterial chemoembolization (TACE), who had similar tumor stage, but better liver function.

In addition to these non-randomized trials, three lager sized RCTs compared treatment with octreotide LAR with placebo in patients with advanced HCC [33,34,35]. In total 460 patients were randomized. In contrast to the uncontrolled trials, no difference in survival was found in any of these studies. The first study by Yuen and colleagues was published in 2002 [33]. Seventy patients with advanced, biopsy proven HCC and untreatable by surgery or TACE, were randomized to receive placebo (*n* = 35) or octreotide LAR (*n* = 35). Treatment consisted in 250 μg short-acting octreotide BID for 2 weeks, followed by octreotide LAR 30 mg IM once every 4 weeks for 6 doses. Overall survival was extremely poor in both groups: 1.93 months versus 1.97 months in the octreotide versus placebo group. In a large multi-center study, 120 patients with advanced biopsy proven HCC were randomly assigned to receive placebo (*n* = 59) or octreotide LAR 30 mg IM once every 4 weeks (*n* = 61) [34]. There was no initial induction with short acting octreotide. Median overall survival time was 4.7 months in the octreotide group compared with 5.3 months in the control group, and 1-year survival was also not significantly different (23 versus 28%). In a large multi-center placebo controlled French study, 272 patients with HCC who were ineligible for curative treatments, were included. Treatment consisted in octreotide LAR, 30 mg IM once every 4 weeks (*n* = 135), or placebo (*n* = 137). Median overall survival was not statistically different in both arms: 6.53 months for octreotide versus 7.03 months for the placebo group. The 1-year survival was similar in both groups (28 versus 30%).

In one study, pasireotide 60 mg IM every 28 days was studied as second line treatment in 20 patients with advanced HCC, of which 55% had metastatic disease [37]. There was no tumor response, and overall survival was nine months. One study evaluated the effect of lanreotide 30 mg IM every two weeks in 21 inoperable patients with advanced HCC. The median survival was 4.2 months. Fifteen of the 21 patients underwent Octreoscan, which was negative in all. However, one patient had a partial response to treatment, whereas, eight patients had stable disease. There were no side effects, on the contrary, quality of life improved in several patients [36]. Moreover, some case reports described treatment success with lanreotide with even disappearance of metastasis in a patient with SSTR2 positive biopsy, but so far, no large scale RCTs using lanreotide for treatment of HCC have been published [38,39].

Several trials have studied additional effects of octreotide when added to other therapies for HCC, including medical treatment, radio frequency ablation (RFA) and surgery. The combination of octreotide LAR 30 mg IM every four weeks and Tamoxifen 20 mg/d was compared to Tamoxifen 20 mg/d in an RCT including 109 patients with advanced HCC [40]. In this study, the association of octreotide LAR with Tamoxifen did not provide any benefit in terms of survival, tumor response or quality of life compared with Tamoxifen alone.

Sorafenib, a multi-kinase inhibitor, was shown to increase overall survival in patients with advanced HCC and preserved liver function [41]. At present, it is the first line treatment for patients with advanced stage HCC and compensated cirrhosis [1]. The combination of octreotide with sorafenib was studied in an uncontrolled trial including 50 patients with advanced stage HCC and Child-Pugh class A or B cirrhosis [42]. The combination was safe and resulted in a median overall survival of 12 months, which was better than historical controls. These findings have to be confirmed in a large RCT before the treatment can be generally advocated.

Twenty-four patients with advanced HCC and Child-Pugh class A cirrhosis were enrolled in an uncontrolled trial evaluating the effect of everolimus 7.5 mg per oral (PO) daily in combination with pasireotide LAR 60 mg IM every 28 days [43]. There was no clear benefit for this combination therapy with a median overall survival of 6.7 months. Treatment was well tolerated, but 25% of patients had hyperglycemia.

Radio frequency ablation is a curative treatment of small HCC lesions. However, when tumor size exceeds 3 cm and/or the number of nodules is >3, the rate of local treatment success is significantly reduced [1]. Therefore, the effect of adding octreotide LAR 30 mg IM every 4 weeks after treatment of HCC with RFA was studied in an uncontrolled trial [44]. All 35 patients were inoperable and had multiple HCC nodules with a maximum diameter of 90 mm; 60% had Child-Pugh class A and 34% Child-Pugh class B cirrhosis. Mean overall survival was 31.5 months and the treatment was well tolerated. This strategy of local treatment followed by octreotide seems to be feasible and safe, even in patients with advanced disease, but needs to be evaluated in a RCT.

In a Chinese study, 99 patients with hepatitis B who underwent curative surgery for HCC, were treated with octreotide LAR 20 mg IM every four weeks for 12 months [45]. Resection specimen and surrounding cirrhotic liver tissue were examined for SSTR2 and five expression at mRNA and protein level. In patients with high expression of SSTR2 and five, tumor recurrence rate was significantly lower as compared with that of patients with low expression (63.83% versus 82.69%). Moreover, overall survival time of the patients with high SSTR expression was longer as compared with survival of patients with low SSTR expression in the tumor. In multivariate analysis SSTR2 expression was an independent prognostic factor for survival. Unfortunately, the study did not include a control group, which precludes firm conclusions in regard of survival benefit of treatment with SST analogues in the postoperative setting.

In a randomized pilot trial, adjuvant lanreotide 40 mg IM once monthly and celecoxib 200 mg BID started before TACE versus TACE alone, was studied in 71 patients with unresectable HCC [46]. The combination therapy significantly prolonged median overall survival (15 versus 7.5 months), enhanced tumor response, and reduced post-embolization syndrome without increase in side effects as compared to TACE alone.

## 4. Discussion

The results of the studies reporting on survival of patients with advanced HCC treated with octreotide analogues are very inconstant. Many studies were small-sized, retrospective and non-randomized. Moreover, only few studies were randomized trials with a sufficient number of patients included.

In some studies, the overall survival was only a few months [33,34], which is less than the expected survival in patients with BCLC advanced stage (C), suggesting that the included subjects had more advanced tumor stage or poor liver function (portal vein thrombosis). Probably, these patients were in bad general health and were selected for the study because they were not fit for other treatments. This subset of patients should not have been included in studies evaluating the effect of octreotide because they will not respond to any treatment. It appears that a minimum of 6 months treatment with SST analogues is needed before a significant clinical benefit can be expected.

Since quality of life is of uttermost importance in this patient population, monthly injections are usually preferred to daily injections. Therefore, one is tempted to use long acting SST analogues. However, from the literature, it seems that long acting SST analogues might be less effective than daily SC injections. In a meta-analysis published in 2011, it was concluded that octreotide could improve the survival of patients with advanced HCC [47]. The results of the meta-analysis showed statistically better survival at 6 and 12 months for patients treated with octreotide. There was no survival difference at 24 months. Further analysis showed that there was a highly statistically significant difference in survival in the studies conducted in China, but not in the studies performed in Western countries. Whether the difference is merely related to ethnicity or other factors was not investigated. However, since short acting SST analogues were used in all trials performed in China and in the sole Western trial that showed survival benefit from octreotide, it is not excluded that this could be a factor in explaining diverse results in different trials. Indeed, when using long acting SST analogues, it takes about three months for steady-state levels to be built up, after which the levels remain rather stable [31]. It is thus probable that patients treated with long acting SST analogues are undertreated during the first few months, during which tumor progression is evident. Therefore, when long acting SST analogues are used, short acting octreotide should be added the first two to three months of treatment. This was not the case in the three large placebo-controlled RCTs [33,34,35]. All three studies were negative, but it is not excluded that the patients were under-treated, explaining the difference with other studies.

Another difference between Chinese and Western studies is the fact that in Chinese studies most patients have chronic hepatitis B infection, whereas in Western trials many patients have alcoholic cirrhosis who might respond less well to treatment with SST analogues [18].

From the clinical trials, it is clear that even if some patients have survival benefit from treatment with SST analogues, others do not. Patient populations and type of treatment were diverse in the different trials, but probably more important is the presence of SSTRs and SSTR subtype expression in the tumor and surrounding tissue. Indeed, only 40–70% of the HCCs express SSTRs, of which not all express SSTR subtypes 2, 3 or 5 for which the synthetic SST analogues have affinity [9,10,11,12,13,14,15]. It makes no sense to treat patients with SST analogues if the tumor does not express SSTRs at all, or only SSTRs for which the SST analogue has no or low affinity. Other reasons for the absence of a significant effect of SST analogues on tumor progression and survival could be resistance to SST analogues, including desensitization of the receptor or the presence of a spliced SSTR5 variant. At present it is unknown if the spliced SSTR5 variant, which blocks SSTR2 signaling and is associated with poor response to SST analogues, is expressed in HCC. In a recent French study, SSTR expression was studied in resection specimen of HCC [11]. It was demonstrated that SSTR2 expression at the membrane of tumor cells was co-expressed with markers of poor prognosis. However, when patients were treated SST analogues after surgical resection of HCC with high expression of SSTR2 and 5, tumor recurrence rate was significantly lower [45].

From studies, it seems that SSTR2, and possible SSTR5, is probably the best target. This could be a reason why pasireotide that has lower affinity for SSTR2 was of no benefit in treating advanced HCC. Therefore, efforts should be made to identify SSTR expression before selecting and starting a SST analogue in a patient with advanced HCC. Ideally, a biopsy of the tumor and surrounding tissue should be performed and examined for SSTR expression. Even if liver biopsy is the gold standard, it is invasive, has side effects and some patients refuse liver biopsy. In a few studies Octreotide scintigraphy was performed before starting treatment with SST analogues [24,31]. In a randomized placebo-controlled trial, in which patients were selected for treatment with octreotide according to scintigraphy positivity, patients had better outcomes in terms of survival and quality of life as compared to a control population [24].

Does the introduction of new systemic treatments (sorafenib, regorafenib, lenvatinib, sunitinib, immunotherapy etc.,) which have proven to be of survival benefit, make SST analogues obsolete in treating HCC? Systemic chemotherapy is restricted to patients with advanced cancer (extrahepatic spread, portal vein invasion), but who have a good liver function (Child-Pugh class A) and performance state ECOG 1-2. Since SST analogues are very well tolerated and have very few side effects, there might be a window of opportunity for patients who are ineligible for chemotherapy, but do not have terminal stage disease for whom all “curative” treatments are probably obsolete, and thus are only eligible for best supportive care. If we want to offer patients a treatment with SST analogues, it should have survival and/or quality of life benefits. Whether SST analogues could have a role for those patients should be evaluated in a prospective randomized placebo-controlled trial, including enough patients to enable demonstrating statistical differences. Primary and secondary end points should be survival, tumor progression and quality of life. As discussed earlier, the patient population should probably consist of patients with advanced disease who have contra-indications or refuse systemic chemotherapy but are not yet candidates for best supportive care.

Should one opt for monotherapy or a combination therapy? Thus far, combination therapy with sorafenib has shown some possible benefit, but at the cost of side effects. An interesting idea is adding SST analogues after RFA, TACE or surgery in patients with high risk of tumor relapse. These strategies were tested in small, mostly uncontrolled trials, but the results were promising [44,45,46]. Efforts should be made to identify SSTR expression on biopsy or resection specimen and in case of high risk, treatment with long acting SST analogues could be of clinical benefit. However, before implementing this treatment strategy, RCTs confirming the results of the uncontrolled studies should be performed.

## 5. Conclusions

Somatostatin analogues have been used with inconsistent success to treat advanced HCC. This could be due to differences in trial design, different dose and type of SST analogues, different patient populations, but also differences in tumor biology including SSTR expression by the tumor or surrounding tissue. From the literature it seems that SST analogues may have a beneficial effect on overall median and 1-year survival in certain populations. In order to identify a specific place for SST analogues in the management of HCC, a placebo-controlled RCT with enough statistical power is needed. It should evaluate the efficacy of SST analogues in patients with advanced, but not terminal stage HCC. Moreover, patients should be selected according to expression of SSTR subtypes, subtypes 2 and 5 being the most promising. Ideally, the selection should be based on identification of SSTRs on liver biopsy specimen, but octreotide scintigraphy might be a good alternative to demonstrate active SSTRs on the cell membrane of tumor cells. If a long acting SST analogue is used, during the first two to three months a short acting analogue should be added in order to ensure high enough plasma levels from the start of treatment. Aside from survival and tumor progression, quality of life is an important secondary end point in this kind of trial. Moreover, a clinical trial assessing the effect of adjuvant therapy of SST analogues after RFA, TACE or surgery in patients with high risk of tumor relapse could be very interesting, as small, mostly uncontrolled trials have been very promising in this setting.

## Figures and Tables

**Table 1:** Trials evaluating somatostatin (SST) Analogues for advanced Hepatocellular Carcinoma.

RCT	Year of Publication	Treatment Regimen	Control	Number of Patients Treated/Control	Median OS (months) Treated vs. Control	1 year Survival (%) Treated vs. Control	Ref.
YES	1998	OCT SC 250 µg BID	NT	28/30	13 vs. 4	56 vs. 13	[23]
YES	2007	OCT SC 500 µg TID, 6 weeks followed by OCT LAR 30 mg IM every 4 weeks	Placebo NT	31/3066 NT	12.3 vs. 77	30 vs. 3	[24]
YES	2001	OCT 200 μg TID	NT	12/13	5.7 vs. 1.6	33 vs. 0	[26]
YES	2003	OCT 200 μg BID	NT	32/33	11.6 vs. 5.6	38 vs. 3	[28]
YES	2004	OCT 100 μg TID	NT	20/25	7 vs. 4	15 vs. 8	[27]
YES	2010	OCT 100 μg TID	NT	21/24	8 vs. 3	38 vs. 8	[29]
YES	2007	OCT 200 μg BID	NT	16/14	7 vs. 2.5	NR	[30]
NO	2002	OCT LAR 20 mg IM every 4 weeks Or LAN 30 mg IM every 2 weeks	historical controls	32/27	15 vs. 8	NR	[25]
NO	2005	OCT 50-250 μg SC TID followed by OCT LAR 30 mg IM every 4 weeks	NO	41/0	19	NR	[32]
NO	2006	OCT LAR 20 mg IM every 4 weeks	NO	63/0	8	38	[31]
YES	2002	OCT 250 μg SC BID for 2 weeks + OCT LAR 30 mg IM every 4 weeks	Placebo	35/35	1.93 vs. 1.97	10.5 vs. 3.3	[33]
YES	2007	OCT LAR 30 mg IM every 4 weeks	Placebo	60/59	4.7 vs. 5.3	23 vs. 28	[34]
YES	2009	OCT LAR 30 mg IM every 4 weeks	Placebo	135/137	6.5 vs. 7.0	28 vs. 30	[35]
NO	2000	LAN 30 mg IM every 2 weeks	NO	21/0	4.3	NR	[36]
NO	2018	Pasireotide 60 mg IM every 4 weeks	NO	20/0	9	NR	[37]

IM: Intramuscular; LAN: Lanreotide; LAR: Long Acting Release; NR: Not Reported; NT: No Treatment; OCT: Octreotide; RCT: Randomized Clinical Trial; SC: Subcutaneously; wks: weeks; TID: thrice daily.

## Abbreviations

BCLC	Barcelona Cancer Liver Clinic
BID	bis in die
ECOG	Eastern Cooperative Oncology Group
HCC	hepatocellular carcinoma
IM	intramuscularly
LAR	long-acting release
PO	per oral
RCT	randomized clinical trial
RFA	Radio frequency ablation
SC	subcutaneously
SST	somatostatin
SSTR	somatostatin receptor
TACE	transarterial chemoembolization
TID	ter in die
